# Glimepiride Administered in Chow Reversibly Impairs Glucose Tolerance in Mice

**DOI:** 10.1155/2018/1251345

**Published:** 2018-10-29

**Authors:** Dana M. Niedowicz, Sabire Özcan, Peter T. Nelson

**Affiliations:** ^1^Sanders Brown Center on Aging, University of Kentucky, Lexington, KY 40536, USA; ^2^Department of Molecular and Cellular Biochemistry, University of Kentucky, Lexington, KY 40506, USA; ^3^Department of Pathology, University of Kentucky, Lexington, KY 40506, USA

## Abstract

Sulfonylureas are a class of antidiabetes medications prescribed to millions of individuals worldwide. Rodents have been used extensively to study sulfonylureas in the laboratory. Here, we report the results of studies treating mice with a sulfonylurea (glimepiride) in order to understand how the drug affects glucose homeostasis and tolerance. We tested the effect of glimepiride on fasting blood glucose, glucose tolerance, and insulin secretion, using glimepiride sourced from a local pharmacy. We also examined the effect on glucagon, gluconeogenesis, and insulin sensitivity. Unexpectedly, glimepiride exposure in mice was associated with fasting hyperglycemia, glucose intolerance, and decreased insulin. There was no change in circulating glucagon levels or gluconeogenesis. The effect was dose-dependent, took effect by two weeks, and was reversed within three weeks after removal. Glimepiride elicited the same effects in all strains evaluated: four wild-type strains, as well as the transgenic *Grn^−/−^* and diabetic *db/db* mice. Our findings suggest that the use of glimepiride as a hypoglycemic agent in mice should proceed with caution and may have broader implications about mouse models as a proxy to study the human pharmacopeia.

## 1. Introduction

The potassium (K^+^) channel subtype, the “K_ATP_ channel,” and its orthologs are expressed in a range of species and have many functions. When K_ATP_ channels are present in the plasma membrane, K^+^ ions are pumped out, thus establishing an ion gradient. Inhibition of K_ATP_ channels induces membrane depolarization and activation of voltage-gated calcium channels. The K_ATP_ channel is also responsive to the ATP/ADP ratio, thus working as a “metabolic sensor” [1]. The channel contains a regulatory subunit, composed of the *ABCC8 or ABCC9 (*ATP-binding cassette, subfamily C members 8 and 9) gene products referred to as sulfonylurea receptor 1 and 2 (SUR1 and SUR2) proteins [2], respectively.

SUR1 and SUR2 are so named because an important drug class, the sulfonylureas, bind to, and block, activity. In the pancreas, sulfonylureas stimulate insulin secretion, leading to their use as oral antidiabetes medication, prescribed worldwide to millions of individuals [3]. Multiple generations of sulfonylurea drugs have been used in the human pharmacopoeia, alone and in drug combinations, varying in their specificity, activity, and impact on human subjects, with much of the variation unexplained to date. Because there has been suggestion that sulfonylurea drugs may have deleterious effects for some persons, this is a topical biomedical issue [4].

Understanding the characteristics of SUR proteins has been a focal point among researchers for decades. The two SUR paralogs are well-conserved across species: protein sequence homology between human and zebrafish SUR2 is ~70% [1]. One reason for evolutionary conservation is that they play phylogenetically durable roles, including metabolism, stress response, and regulation of blood vessel function [2, 5–9]. *SUR1* and *SUR2* allelic variants are associated with human diseases, including congenital diabetes, heart disease, and CNS disorders [6]. SUR1 function is best understood in the vertebrate pancreas and central neuronal tissue, as a regulator of insulin secretion and neuronal excitability.

Rodent studies are a common experimental context in which to study SUR physiology and pharmacology. A PubMed search combining “Sulf/phonylurea” and "rat/mouse" returns over 5400 published papers. However, there has been some indication that the pharmacological effect of sulfonylureas seen in humans differs appreciably from that seen in rodents in lab experiments. It is important to understand such a discrepancy, both for relevance to experimental models and for understanding the pharmacobiology of drugs commonly used in humans.

Here, we report the results of studies using the orally-administered sulfonylurea drug glimepiride (tradename Amaryl™) in six different mouse strains, in order to understand how the drug affects glucose homeostasis and tolerance. We used glimepiride sourced from a local pharmacy for comparison to human clinical use. Contrary to expectations, glimepiride exposure was associated with increased blood glucose levels and impaired glucose tolerance in all strains tested, both of which were reversible after several weeks wash-out.

## 2. Materials and Methods

### 2.1. Animals

All animal work was conducted with prior University of Kentucky IACUC approval and was performed in accordance with USDA and PHS guidelines. Information about the mouse (*Mus musculus*) strains used, including age, length of treatment, and tests performed, is summarized in Table 1. All strains were obtained from the Jackson Labs (C57Bl/6J, C57Bl/6N, BalbC, and C3H; Bar Harbor, ME) or in-house breeding colonies at the University of Kentucky (*Grn^−/−^* [10, 11] and *db/db*). *db/db* mice were on a hybrid C57Bl/6J/CD-1/129 background, described previously [12]. Mice were group housed, fed and provided with water *ad libitum*, and maintained on a constant 12-hour light/dark cycle. Glimepiride was obtained by prescription and milled into chow (1 or 8 mg/kg/day: BioServ Custom Diet:, Flemington, NJ). We based our estimate of glimepiride dose on a 25 g mouse, and an average food consumption of 5 g per day. Nicorandil (Tocris: Minneapolis, MN; TCI, Portland, OR) was administered in drinking water (15 mg/kg/day), based on an average of 5 mL of water consumed per day. Control mice were fed a control diet (BioServ) with a consistent nutrient content and given control water with no additives. For the wash-out experiment, mice were tested three weeks after removal of glimepiride chow. Mice were euthanized by CO_2_ asphyxiation, followed by decapitation, and the liver and serum frozen until use.

### 2.2. Metabolic Testing

Mice were tested using standard glucose [13], insulin [14], or pyruvate [15, 16] tolerance testing. For glucose and insulin tolerance, mice were fasted for six hours in advance of testing. A drop of blood was obtained by tail nick, and the baseline blood glucose was measured by glucometer (Bayer Breeze 2: Bayer, Tarrytown, NJ). Mice were then injected intraperitoneally with glucose (2 mg/g body weight: Vedco, St. Joseph, MO) or insulin (0.75 U/kg body weight; Humulin, Eli Lilly, Indianapolis, IN). Blood glucose was tested at multiple time points over two to three hours. Any measurement giving a reading of “HI” on our glucometer was set to 700 mg/dL for analysis. For pyruvate tolerance testing, mice were fasted overnight (16–18 hours) in order to deplete glycogen stores. After a baseline glucose measurement, pyruvate (2 mg/g body weight; Sigma-Aldrich, St. Louis, MO) was injected intraperitoneally and blood glucose was assessed over three hours.

### 2.3. Serum Hormone Immunoassays

Serum was obtained by either saphenous bleed or trunk bleed (at euthanasia). Insulin and glucagon serum levels were analyzed using mouse-specific, commercially available ELISA (Insulin, EMD Millipore, Billerica, MA) or EIA (Glucagon, Sigma-Aldrich) kits, according to manufacturers' instructions.

### 2.4. Glycogen Measurement

Liver glycogen was extracted as reported [17, 18]. Briefly, livers were homogenized in 10 volumes 30% KOH, using a Polytron homogenizer (PRO200: ProScientific, Oxford, CT), then heated at 95°C for 30 minutes. Samples were cooled on ice, then 1.5 volume of 95% ethanol was added. Samples were centrifuged (3000 ×g, 20 minutes at 4°C) to precipitate glycogen. The supernatant was discarded, and the pellet was washed with 1 volume water and 1.5 volume 95% ethanol. The resulting pellet was dissolved in 1 volume water. The extracted glycogen was reacted with anthrone reagent (Sigma Aldrich: 14.6 mM anthrone in concentrated H_2_SO_4_) for 20 minutes at 90°C [17, 18]. Absorbance was detected at 625 nm and compared against a standard curve of D-glucose.

### 2.5. Data Analysis

Measurements from each mouse were counted as individual data and then used to calculate group averages. All data are reported as group mean ± SEM. For simplification, GTT data is expressed as the area under the curve (AUC) for the time course, though comparisons at each time point were also made. Data were analyzed with SPSS (Hewlett Packard, Palo Alto, CA) using the general linear model (GLM) module for ANOVA with the independent variables (as needed) treatment, genotype, and fasting state (for explanation of this model, http://pic.dhe.ibm.com/infocenter/spssstat/v21r0m0/index.jsp?topic=%2Fcom.ibm.spss.statistics.help%2Fidh_glm_multivariate.htm).

## 3. Results

### 3.1. Glimepiride Treatment Causes an Impairment in Glucose Tolerance

**Figure 1:**
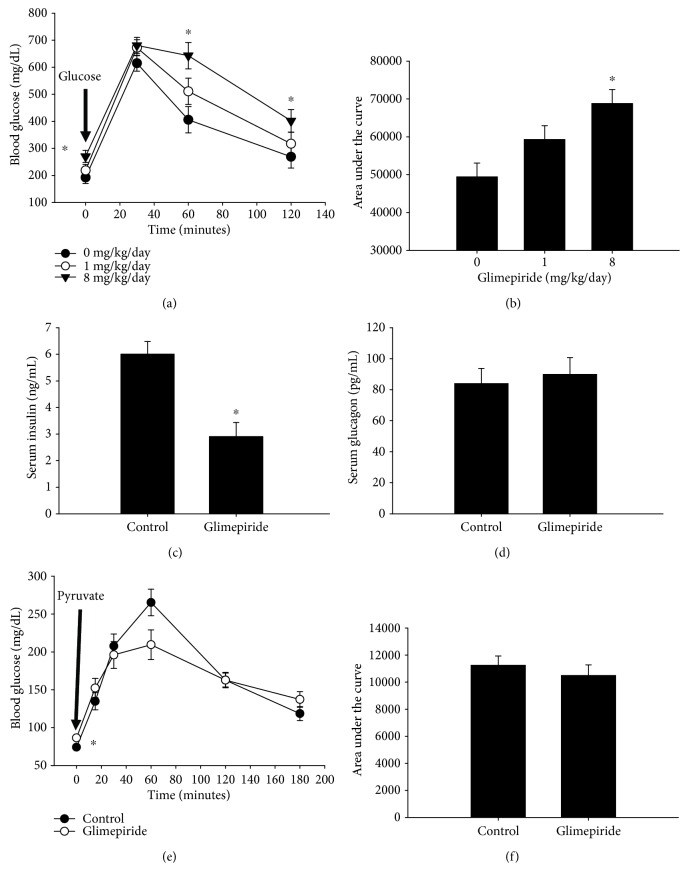
Glimepiride treatment impairs glucose tolerance in C57Bl/6J mice. Wild-type C57Bl/6J mice were treated for two weeks with glimepiride (1 or 8 mg/kg/day) or control chow, after which a glucose tolerance test (GTT) was performed. Blood glucose was measured after a 6-hour fast (time = 0). The mice were then injected with glucose (2 mg/g body weight), and the blood glucose was measured over the next 2 hours. Mice treated with 8 mg/kg/day glimepiride had significantly elevated fasting blood glucose and higher blood glucose at *T* = 60 and 120 minutes (a) (^∗^*p* ≤ 0.04). Closed circles = control group; open circles = 1 mg/kg/day; inverted triangles = 8 mg/kg/day glimepiride. The area under the curve was also calculated for the GTT time course as a representation of glucose tolerance. Mice treated with 8 mg/kg/day glimepiride had significantly impaired glucose tolerance (bigger area under the curve) than control animals (b) (^∗^*p* ≤ 0.001). Treatment with 1 mg/kg/day glimepiride trended toward impaired glucose tolerance, though this did not reach significance (*p* ≤ 0.07). *n* = 6/group. Circulating insulin (c) and glucagon (d) levels were measured after glimepiride treatment (8 mg/kg/day; *n* = 6/control, *n* = 5/glimepiride). Serum insulin was decreased by approximately 50% in mice treated with glimepiride (^∗^*p* ≤ 0.002). Unlike insulin, serum glucagon was unaffected by glimepiride treatment. Gluconeogenesis was assessed using a pyruvate tolerance test. Even after an overnight fast, glimepiride-treated mice had elevated fasting blood glucose (e) (*p* < 0.02). However, there was no difference in blood glucose in any of the other time points, or in the area under the curve (f). *n* = 6/control; *n* = 5/glimepiride. Closed circles = control diet; open circles = glimepiride group.

In order to minimize stress to the animals, we chose to administer glimepiride in chow. Wild-type C57Bl/6J mice were fed *ad libitum* with glimepiride chow for two weeks, after which a glucose tolerance test was performed. Glimepiride was well-tolerated, with no significant adverse complications , including no observed hypoglycemic events. Glimepiride treatment did not cause a change in weight (*not shown*). Contrary to published reports, glimepiride treatment increased fasting blood glucose and blood glucose at most of the time points after glucose injection (Figure 1(a)), at least at 8 mg/kg/day. There was also an increase in the area under the curve for the time course, indicative of impaired glucose tolerance (Figure 1(b)). The lower dose (1 mg/kg/day) trended toward an increase in the area under the curve (*p* = 0.07).

Sulfonylureas, such as glimepiride, are widely used in human diabetic patients to stimulate insulin secretion from pancreatic beta cells and subsequently reduce hyperglycemia. In order to determine the effect of glimepiride on insulin secretion in mice, we measured serum insulin by ELISA. The nonfasting serum insulin concentration was reduced by half in mice treated with glimepiride (Figure 1(c)). This suggests that glimepiride is acting on the pancreatic beta cells to reduce, instead of promote, insulin secretion.

In order to maintain a constant level in between and after meals, blood glucose is highly regulated. This is accomplished primarily through a combination of hormonal and neural control of cellular processes. In addition to insulin, the pancreatic hormone glucagon regulates many aspects of cellular metabolism, especially during fasting. We, therefore, determined the effect of glimepiride on the secretion of glucagon. Glimepiride treatment had no effect on serum glucagon levels (Figure 1(d)), indicating that the hormone is not a major target of glimepiride action.

The liver is also a major driver of glucose homeostasis, both for glucose generation during fasting as well as glucose uptake and long-term storage. An increase in gluconeogenesis in the liver could explain higher fasting blood glucose in glimepiride-treated animals. We performed a pyruvate tolerance test after overnight fasting in order to deplete liver glycogen stores. Even after fasting overnight, mice treated with glimepiride had significantly elevated blood glucose compared with untreated animals (Figure 1(e)). This may indicate the presence of residual glycogen. There was no difference in glucose production after pyruvate challenge, however (Figures 1(e) and 1(f)), indicating that there was no stimulation of gluconeogenesis due to glimepiride treatment.

**Figure 2:**
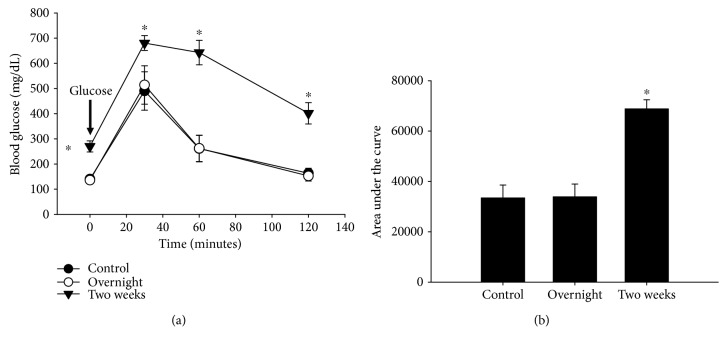
Overnight glimepiride treatment has no effect on glucose tolerance. C57Bl/6J mice were treated overnight or for 2 weeks with glimepiride (8 mg/kg/day), then glucose tolerance was assessed. Unlike the 2-week treatment, overnight treatment with glimepiride (8 mg/kg/day) had no effect on either fasting blood glucose (a) or glucose tolerance (b). ^∗^*p* ≤ 0.04; *n* = 3/control, *n* = 3/overnight, *n* = 6/2 weeks. Closed circles = control group; open circles = overnight treatment; inverted triangles = two-week treatment.

The changes in insulin output and glucose tolerance occurred after two weeks of treatment, far shorter than the time frame that human diabetics are typically prescribed glimepiride. We reasoned that glimepiride might initially stimulate insulin secretion, which becomes suppressed over time, possibly due to feedback inhibition. To determine whether there was an improvement in glucose control after a very short-term treatment, we performed an acute, overnight treatment with glimepiride (8 mg/kg/day). Overnight treatment did not elicit an increase in fasting blood glucose or an impairment in glucose tolerance (Figures 2(a) and 2(b)), unlike the two-week treatment. However, we did not see a *decrease* in fasting or post-injection glucose levels, as previously described [19].

### 3.2. Glimepiride Treatment in Different Strains

**Figure 3:**
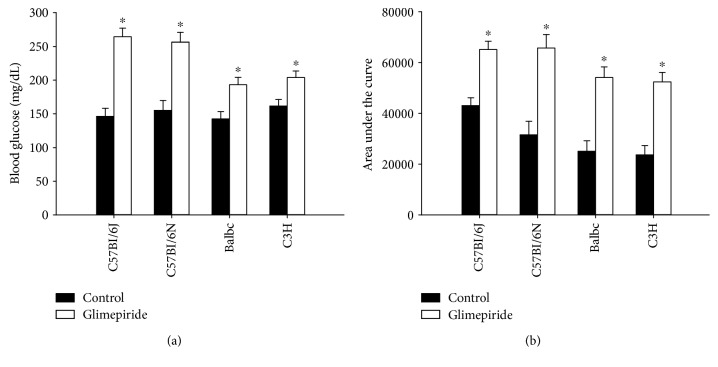
Glimepiride impairs glucose tolerance in many different strains. We treated a variety of wild-type inbred mice with glimepiride (8  mg/kg/day) or control chow for 2 weeks, then performed a glucose tolerance test. C57Bl/6J (*n* = 6/control, *n* = 5/glimepiride), C57Bl/6N (*n* = 6/group), BalbC (*n* = 5/group), and C3H (*n* = 6/group) mice showed both elevated fasting blood glucose (a) and impaired glucose tolerance (b) when treated with glimepiride. ^∗^*p* ≤ 0.02 compared with control animals. Black bars = control group; white bars = glimepiride group.

C57Bl/6J mice are known to have a predisposition to obesity and diabetes [20, 21]. We wondered, therefore, if the effect on glucose tolerance was limited to C57Bl/6J mice or was a more universal phenomenon. To test this possibility, we treated three additional, commonly-used inbred mouse strains with glimepiride (8 mg/kg/day) for two weeks: C57Bl/6N (a related substrain without the susceptibility to diabetes [20]), BalbC, and C3H mice. The strains differed with respect to their glucose tolerance in the absence of glimepiride: C3H and BalbC mice had the best glucose tolerance (smallest area under the curve), while C57Bl/6J had the worst (Figure 3(b)). Glimepiride treatment caused a significant increase in fasting blood glucose (Figure 3(a)) in all strains tested, though the C57Bl/6 substrains displayed the largest increase (70 vs. 30%). Glucose tolerance was also impaired in glimepiride-treated animals of all strains tested (Figure 3(b)).

**Figure 4:**
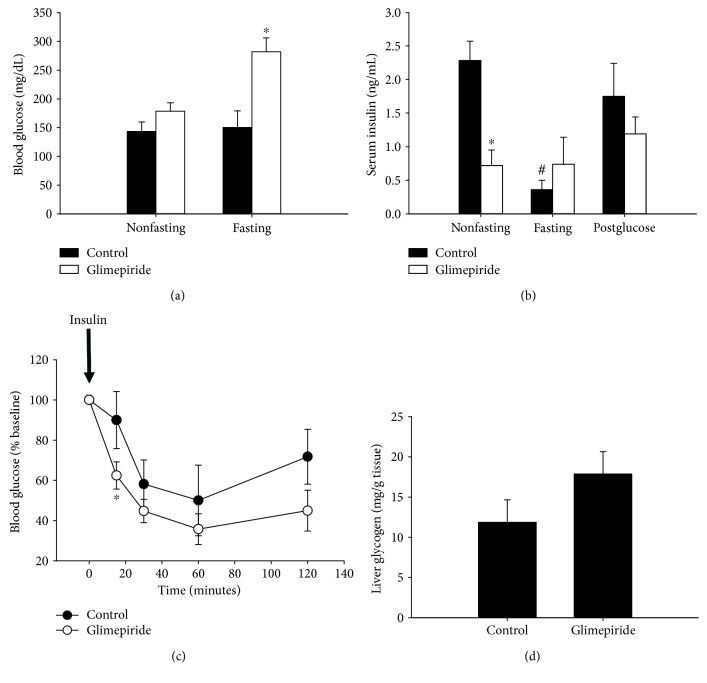
C57Bl/6N mice have decreased insulin, but normal insulin tolerance in response to glimepiride (8 mg/kg/day) treatment. Mice were fasted for 6 hours prior to the start of the GTT. There was no difference in nonfasting blood glucose between control and glimepiride-treated mice (a). Control-fed mice maintain a constant blood glucose levels during this fast. Glimepiride-fed mice, on the other hand, have increased blood glucose after the 6-hour fast. We also tested insulin levels before and after fasting. Even in nonfasting animals, glimepiride treatment decreases circulating insulin levels (b). Insulin decreases in control-fed mice during the fasting period, but does not change in glimepiride-fed animals. Administration of a glucose bolus stimulated insulin secretion in control-fed animals, but not in glimepiride-fed animals. Black bars = control group; white bars = glimepiride group (8 mg/kg/day). In order to measure insulin sensitivity, we performed an insulin tolerance test. Glimepiride-treated mice are as sensitive to insulin as control-treated mice (c). In fact, glimepiride-treated mice have lower blood glucose 15 minutes after insulin injection. Closed circles = control group; open circles = glimepiride group. Liver glycogen levels were not significantly different in glimepiride-treated mice, though there was a trend towards an increase *p*-value of < 0.2 (d). *n* = 6/group: ^∗^*p* ≤ 0.03.

The factors that regulate blood glucose in the fed state differ from those in the fasting state (e.g., insulin vs. glucagon). Therefore, we also examined the difference between nonfasting and fasting blood glucose in C57bl/6N mice. Though there was not a significant difference between treatment groups in nonfasting mice, blood glucose increased nearly two-fold in glimepiride-treated mice during the six-hour fasting period (Figure 4(a)). Untreated mice maintained constant blood glucose during that time. Similarly, we measured serum insulin in the nonfasting and fasting period. Control mice displayed a significant decrease in serum insulin during the fast (Figure 4(b)), consistent with the lack of food intake. As observed in C57Bl/6J mice, glimepiride-treated C57Bl/6N mice had significantly lower serum insulin in the nonfasted state than control-treated mice. A six-hour fast did not change insulin levels in treated mice. We also measured serum insulin 30 minutes after i.p. administration of glucose, as performed during a GTT (approximately the time at which blood glucose peaks). Glucose administration stimulated insulin secretion in the untreated mice, but did not significantly change insulin levels in glimepiride-treated mice (Figure 4(b)), indicating that glimepiride treatment not only suppresses insulin secretion in the fed state but also in response to a glucose bolus.

These data suggest that (1) glimepiride-treated animals are able to maintain normal blood glucose under fed conditions, despite low insulin levels, and (2) a change in circulating insulin is not responsible for the increase in blood glucose during the fasting period. Insulin's primary target tissues are muscle and liver, which take up blood glucose for acute use as well as long-term storage. To determine whether glimepiride impacts insulin sensitivity, we performed an insulin tolerance test on C57Bl/6N mice. Both treated and control mice were sensitive to insulin, showing a time-dependent decrease in blood glucose after insulin administration (Figure 4(c)). In fact, glimepiride-treated mice had lower blood glucose at 15 minutes after insulin injection, indicating that they may be more sensitive to insulin action than untreated mice. We also postulated that an increase in glycogen storage could account for the increase in fasting blood glucose observed in glimepiride-treated mice. We measured glycogen levels in the liver since it is a primary site of storage and is partially responsible for the maintenance of blood glucose availability during fasting. There was a trend toward elevated glycogen in glimepiride-treated mice, though this did not reach significance (Figure 4(d), *p* < 0.2). This increase was mostly observed in male mice (*not shown*).

### 3.3. Reversibility of the Glimepiride Effect

**Figure 5:**
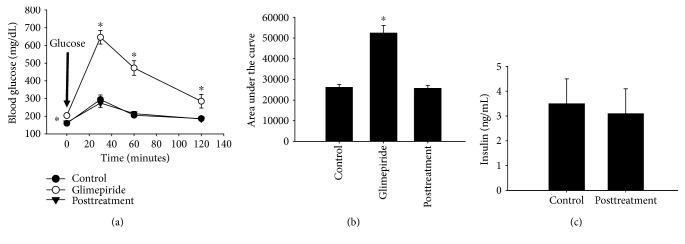
The effect of glimepiride on glucose tolerance is reversible. C3H mice were treated with glimepiride (8 mg/kg/day) for 2 weeks, after which glucose tolerance was assessed. The mice were then switched to the control diet for 3 weeks, and the glucose tolerance was reassessed. After 2 weeks of glimepiride treatment, blood glucose was elevated after fasting and at every time point during the GTT (a). Three weeks after removal of glimepiride, glucose levels had returned to control levels. The area under the curve was increased in glimepiride-treated animals, but returned to normal by three-week posttreatment (b). By three-week posttreatment, insulin levels had also normalized (c). *n* = 6/group. ^∗^*p* ≤ 0.0005. Closed circles = control group; open circles = glimepiride group (two-week treatment); inverted triangles = three-week posttreatment.

We next wanted to know if the effect on glucose tolerance and impairment of insulin secretion was a long-term deficiency, which would indicate permanent *β*-cell damage, or would return to normal upon removal of the drug. We treated wild-type C3H mice with glimepiride for two weeks, then switched them to control diet for three weeks in order to affect a systemic “wash out” of the drug, after which we performed another GTT. Wild-type C3H mice treated with glimepiride had significantly elevated fasting glucose and impaired glucose tolerance after a two-week treatment (Figures 3, 5(a), and 5(b)). These impairments returned to normal after the drug was removed (Figures 5(a) and 5(b)), indicating that the glimepiride-induced changes in glucose tolerance were not permanent. Similarly, insulin levels in the 3-week post-treatment mice were not significantly different from control animals (Figure 5(c)).

### 3.4. Glimepiride Treatment in Progranulin Knockout Mice

**Figure 6:**
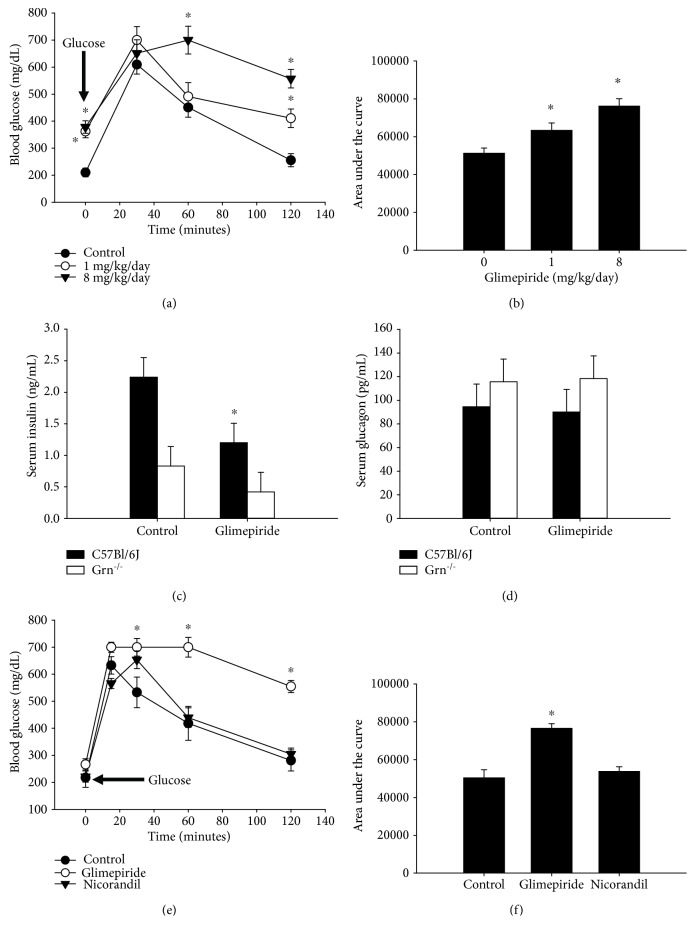
Glimepiride impairs glucose tolerance and suppresses insulin secretion in *Grn^−/−^* mice. *Grn^−/−^* mice were treated with glimepiride (0, 1, or 8 mg/kg/day) for two weeks, after which a glucose tolerance test was performed. Both doses of glimepiride led to an increase in fasting blood glucose in these mice (a) (*n* = 12/control, *n* = 6/1 mg/kg/day, *n* = 6/8 mg/kg/day). Closed circles = control; open circles = 1 mg/kg/day glimepiride; inverted triangles = 8 mg/kg/day glimepiride. In addition, both doses increased blood glucose during at least part of the GTT, leading to an increase in the area under the curve (b). Serum insulin was lower in *Grn^−/−^*mice than wild-type (c) (*n* = 5/group). Glimepiride (8 mg/kg/day) treatment significantly reduced circulating insulin in wild-type mice, but there was no change in *Grn^−/−^* mice. Note that any samples that were below the detection limit for the insulin ELISA were set to 0 and included in the group mean. Glucagon was unaffected by both genotype and glimepiride treatment (d) (*n* = 5/group). Black bars = C57Bl/6J wild-type; white bars = *Grn^−/−^* mice. We tested nicorandil (15 mg/kg/day), another compound that targets sulfonylurea receptors, but is specific for SUR2. Unlike glimepiride, nicorandil treatment had no effect on fasting blood glucose (e) or glucose tolerance (f) (*n* = 2/control, *n* = 6/glimepiride, *n* = 6/nicorandil). ^∗^*p* ≤ 0.02. Closed circles = control; open circles = glimepiride (8 mg/kg/day); inverted triangles = nicorandil.

Progranulin knockout mice (*Grn^−/−^*) have been used to model neurodegenerative diseases, such as frontotemporal dementia [10, 22, 23]. Progranulin and the subsequently-produced granulins play distinct roles in inflammation and lysosomal function [10, 24–27]. Circulating progranulin has been positively correlated with diabetes [28–30], with *Grn^/−^* mice protected from high-fat diet-induced diabetes [31]. It is possible, therefore, that *Grn^−/−^* mice would be resistant to glimepiride-induced changes in glucose control. In order to test this hypothesis, we treated *Grn^−/−^* mice (on a C57Bl/6J background) with glimepiride (1 or 8 mg/kg/day) for two weeks, then assessed glucose tolerance. Fasting blood glucose was elevated at both doses, and blood glucose levels were high throughout the assay (Figure 6(a)). Even the lowest dose tested (1 mg/kg/day) impaired glucose tolerance, as evidenced by the increase in the area under the curve (Figure 6(b)). Interestingly, serum insulin was significantly lower in untreated *Grn^−/−^* mice, compared with age-matched wild-type C57Bl/6J mice (Figure 6(c)). There was a trend toward reduced insulin levels in glimepiride-treated *Grn^−/−^* mice, though this did not reach significance. In many cases, insulin levels were below the assay detection limit in glimepiride-treated *Grn^−/−^* mice (note that these were included in the group mean that was displayed in the graph). Glimepiride treatment did not affect glucagon levels (Figure 6(d)).

Sulfonylureas maintain K_ATP_ channels in the closed position by binding to the regulatory sulfonylurea receptor and promoting insulin secretion. Glimepiride, in particular, has specificity for both the pancreatic SUR1 and SUR2, an isoform present in other cell types, such as cardiac muscle [3]. We next asked whether modulation of SUR2 could elicit an effect on glucose. In order to test this, we treated *Grn^−/−^* mice with nicorandil, a SUR2 modulator used clinically to maintain K_ATP_ channels in the open position, thus preventing membrane depolarization. Unlike glimepiride, nicorandil treatment did not alter either fasting blood glucose or glucose tolerance (Figures 6(e) and 6(f)), suggesting that SUR2 agonism has no effect on glucose tolerance. However, it is still unclear if the effect of glimepiride is solely due to SUR1 binding or could involve SUR2 in extrapancreatic tissues.

### 3.5. Glimepiride Treatment of *db/db* Mice

**Figure 7:**
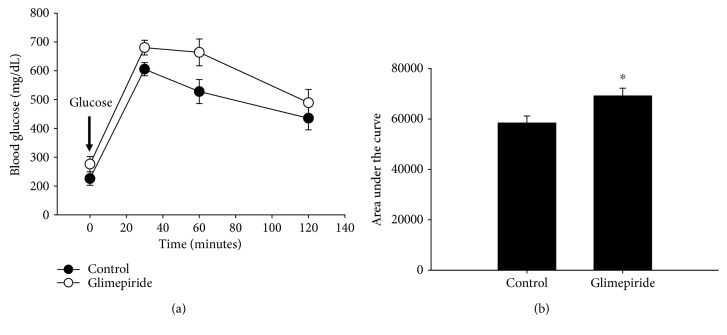
Glimepiride impairs glucose tolerance in *db/db* mice. We treated the obese, insulin-resistant *db/db* mouse with glimepiride (1 mg/kg/day). Though blood glucose was not significantly affected at any of the time points during the GTT (a), there was an overall impairment in glucose tolerance (b). *n* = 6/control, *n* = 5/glimepiride. ^∗^*p* ≤ 0.04. Closed circles = control; open circles = glimepiride.

Diabetic mice, such as the obese, insulin-resistant *db/db* strain [12], are used extensively in research and are often treated with antidiabetic drugs, such as sulfonylureas, to mimic the relevant human clinical course. It is, therefore, of interest to know whether these drugs are having the intended effect in the employed models. We, therefore treated *db/db* mice with glimepiride (1 mg/kg/day) for two weeks. Untreated *db/db* mice are already hyperglycemic and glucose intolerant, as compared to wild-type animals (Figures 1(a), 7(a), and 7(b), [12]). Glimepiride treatment further exacerbated these deficits, even at the relatively low dose used in this study. Therefore, despite already existing metabolic derangements, *db/db* mice are still susceptible to the effects of glimepiride treatment.

## 4. Discussion

Sulfonylureas have been widely used as insulin secretagogues to reduce hyperglycemia in human diabetic patients, as well as research animals. In spite of the reported success of these treatments in humans, at least in regard to glycemic control, robust translation to rodent models is unclear. In order to test the impact of sulfonylurea medications in mice, we treated a variety of strains with the third-generation sulfonylurea glimepiride, using a preparation that was generated for the purpose of treating human diabetic patients and sourced from a local pharmacy. We found that, contrary to expectations, glimepiride exposure was associated with inhibition of insulin secretion, increased fasting glucose levels, and an impairment in glucose tolerance. These effects required more than a day to take effect, with impairment of glucose tolerance by two weeks, and were seen in all strains tested including mice that are reportedly resistant to diabetes (*Grn^−/−^* mice [29]) and those with impairments in glycemia and insulin sensitivity (*db/db* mice [12]). Indeed, among the dozens of mice tested, we observed no hypoglycemic episodes and no reduction in blood glucose after exposure to the glimepiride. The negative effects of glimepiride on blood glucose levels and glucose tolerance were reversed by several weeks of “wash-out,” indicating that the induced changes are not permanent.

The rise in blood glucose during the six-hour fasting period is an interesting finding and is likely independent of the decrease in circulating insulin, which does not significantly change in glimepiride-treated animals during fasting. There was no increase in gluconeogenesis in glimepiride-treated mice, nor did glucagon levels change. There was a trend toward increased liver glycogen in glimepiride-treated animals, which may account for the surge in blood glucose. This would be consistent with published findings [32].

There are several caveats to these data: (1) All treatments used for glucose tolerance testing were performed in one vivarium, raising the possibility of a locally specific effect. We feel that this is unlikely, both due to the consistency between various strains and due to the fact that we did observe the decrease in insulin levels in mice treated in a separate vivarium. (2) Likewise, we used glimepiride from a single source to be milled into feed. Given the amount of time over which these experiments were performed (~3 years), and the number of batches of feed generated (4+), we feel that source bias is unlikely to be a contributing factor in our results. In addition, we also performed small pilot experiments where we administered glimepiride in drinking water (*not shown*; the glimepiride in these experiments was sourced from the apothecary at the University of Kentucky). (3) Although we measured serum insulin in response to fasting or glucose bolus, as well as during the nonfasting period, we did not determine if this was due to the downregulation of insulin production or a loss of secretion itself.

We focused on glimepiride for these studies, both for its popularity among human diabetics and our interest in SUR2 modulation specifically. Sulfonylureas have been used for over half a century to control hyperglycemia, and prescriptions still top 80 million per year in the U.S. [33]. Glimepiride is the most commonly prescribed sulfonylurea, due in part to a lower instance of acute hypoglycemic episodes and reduced cardiovascular complications. Glimepiride is somewhat unique among the sulfonylureas for its dual specificity for SUR1 and SUR2 [3], raising the possibility of effects in multiple tissues.

Glimepiride and other sulfonylureas have been used extensively in rodent studies in order to study their effects on the pancreas and other organs. These studies vary widely with regard to the rodent models tested (including both wild type and diabetic rodents), as well as the dose and the length of treatment (Table 2). The parameters tested in this study encompass both the published dose ranges (1–8 mg/kg/day) and treatment length (1 day to 2 weeks). Despite aligning with reported methods, our results contradict the prevailing dogma that glimepiride reduces blood glucose by stimulating insulin secretion. One difference of note is that published studies predominantly use a once daily administration, by either oral gavage or intraperitoneal injection. In contrast, we delivered glimepiride in chow (or drinking water in a limited number of experiments). Chow administration has the advantage of minimizing stress to the animals, as well as providing a route of absorption similar to that of humans ingesting a pill (as opposed to injection). However, the mice eat multiple times per day and, thus, would receive the indicated dose spread out over a day, versus a single bolus. This may have implications on the steady-state level of glimepiride.

The results of this study were quite surprising given the described mode of sulfonylurea action, leading us to repeat the experiments with multiple doses of glimepiride and testing six different strains of mice. Glimepiride treatment elicited the same effect on blood glucose in all strains tested, including both wild type and transgenic animals, leading us to conclude that this phenomenon is more universal to rodents. The direct relevance to humans is less clear.

The specific mechanistic pathway(s) underlying these results are yet to be fully elucidated. Prior studies, primarily in cell culture, indicate that the first generation sulfonylureas induce a decrease in both *β*-cell granularity and insulin secretion, though the mechanism underlying these changes is unclear [34]. A prior study treating mice with glibenclamide showed higher blood glucose levels after a week of treatment [35]. Less is known about the changes induced by glimepiride specifically, though it is known to have extrapancreatic targets which may also affect glucose regulation, including adipocytes and the hypothalamus [19]. One recent study showed that glimepiride treatment alone had no effect on fasting blood glucose or glucose tolerance in ZDF rats and postulated that peroxynitrite-induced *β*-cell damage could explain this phenomenon, though these investigators did not test the hypothesis [36]. Peroxynitrite is very reactive and challenging to study experimentally, but this could be an avenue for future research.

Though many patients on sulfonylurea mono- or multidrug therapy eventually experience *β*-cell failure and must be treated with insulin, the clinical course of these drugs can extend to decades of use [4]. There is some evidence that this failure may not be due to sulfonylurea treatment itself, but rather a loss of function due to years of diabetic insult (hyperglycemia) [37–39]. In addition, some sulfonylureas are more protective against *β*-cell failure than others [4].

Our results show that suppression of insulin secretion occurs in mice after only two weeks of glimepiride treatment. There are a large number of hypothetical explanations that could explain our observations. We highlight four possibilities: (1) sequence and/or structural differences in SUR1 that would affect glimepiride-mediated inhibition of K_ATP_ channels, (2) desensitization of voltage-gated calcium channels to membrane depolarization, (3) species-specific differences in pathways involved in insulin biosynthesis and storage, which would result in a decrease in *β*-cell insulin granules, and (4) in this study, we administered glimepiride in chow, while humans take a pill once a day. This may have repercussions in regard to steady-state levels, versus a spike in circulating levels that decreases before the next dose, causing a more sustained activation of voltage-gated calcium channels.

## 5. Conclusions

In conclusion, short-term (two weeks or less) glimepiride treatment in mice caused significant impairment of insulin secretion, resulting in profound glucose intolerance. This includes a substantial increase in fasting blood glucose, though insulin sensitivity was unimpaired. Whatever the underlying mechanism(s), it is not entirely clear that it is independent of what occurs in humans. Our findings suggest that the use of sulfonylureas as a hypoglycemic agent in mice should proceed with caution and may have broader implications about the use of mouse models to study the human pharmacopeia.

## Figures and Tables

**Table 1:** Mouse strains and treatments.

Strain	*N*	Assay	Treatment length	Age (months)
C57Bl/6J	29	GTT	2 weeks	2.5–5
	11	PTT	1.5 months	3.4
	12	ELISAs	1.5 months	3.5

C57Bl/6N	12	GTT	2 weeks	3.7
	11	ITT	1 month	4.2
	12	ELISAs	1.5 months	4.8

C3H	12	GTT	2 weeks	2.4
	12	GTT^†^	3 weeks	3
	12	ELISAs	‡	3.1

BalbC	10	GTT	2 weeks	2.4

*Grn^−/−^*	18	GTT	2 weeks	5–10
	10	ELISAs	8 months	21

*db/db*	11	GTT	2 weeks	4.4

^†^Performed after three weeks after removal of glimepiride (wash out). ^‡^Blood was collected at endpoint, soon after the three-week wash out.

**Table 2:** Rodent glimepiride treatments in previous publications.

Species	Strain	Glimepiride (mg/kg/day)	Route of administration	Length of treatment	Blood glucose	Reference
Mouse	*ApoE^−/−^*	0.1	Oral gavage	8 weeks	Decreased	[40]
	KK-Ay	0.5	Oral gavage	8 weeks	Decreased	[41]
	C57Bl/6	2	Oral gavage	4 weeks	Decreased	[42]
	*db/db*/C57Bl/6 + STZ	2.5	Oral gavage	6 weeks	Decreased	[43]
	*KsJ-db/db*	2.5	Oral gavage	4 weeks	Decreased	[44]
	BALB/c	4	i.p.	3 weeks	Decreased	[45]
	Swiss albino + STZ	10	Oral gavage	1 week	Decreased	[46]

Rat	OLETF	0.05	Chow add-mixture	12 weeks	Unchanged	[47]
	Wistar ± STZ	0.09	Oral gavage	<1 day	Decreased	[48]
	Wistar	0.1	Drinking water	4 weeks	n.d.	[32]
	Sprague-Dawley	4	Oral gavage	3 days	n.d.	[49]
	Wistar	5	i.p.	2 days	Unchanged	[50]
	ZDF	5	Oral gavage	12 weeks	Unchanged	[36]
	BB	200	Oral gavage	108 days	Decreased	[51]
	Wistar ± STZ	200	Oral gavage	3 days/8 weeks	Decreased	[52]

i.p. = intraperitoneal injection; n.d. = not determined; OLETF = Otsuka Long-Evans Tokushima fatty; STZ = streptozotocin; ZDF = Zucker diabetic fatty.

## Data Availability

The data used to support the findings of this study are available from the corresponding author upon request.
